# The Association between Comorbidities and Cognitive Function: Findings from the Health and Retirement Study

**DOI:** 10.1002/alz.088273

**Published:** 2025-01-09

**Authors:** Yan‐Jhu Su, Andrew Alberth, Alison C Rataj, Elisabeth Stam

**Affiliations:** ^1^ University of Massachusetts Boston, Boston, MA USA

## Abstract

**Background:**

Cognitive function has been linked to factors including physical health and individual characteristics (e.g., race, ethnicity). However, few studies have examined these associations simultaneously. Therefore, this study examined the relationship between chronic conditions and cognitive function, and whether this differed for people across race and ethnicity among adults 65 years old and older in the United States.

**Method:**

This cross‐sectional study uses data from the 2018 Health and Retirement Study (HRS). The sample includes adults, age 65 years and older, who were employed in 2018 (*N* = 8,310). The main dependent variable is respondents’ cognitive function (TICS‐35) in 2018 and the independent variable is the different chronic illness conditions and chronic illness count. Race and ethnicity are assessed as moderators. Ordinary least squares (OLS) regression models are utilized in this investigation.

**Result:**

The results show that individuals with chronic lung disease such as chronic bronchitis or emphysema (b = ‐.56, *p* = .001), stroke (b = ‐.56, *p*<.001), or psychological issues (b = ‐.71, *p*<.001) had lower cognitive function scores than other individuals. Moreover, racial and ethnic differences were observed, specifically older Black adults with psychological issues scored lower on cognitive function scores when compared to White older adults (b = ‐.73, *p* = .075). Older adults with more chronic illnesses had a lower cognitive function score (b = ‐.11, *p* = .01) and racial and ethnic differences were observed as well, specifically older Hispanic adults with more chronic illness had lower cognitive function than White older adults (b = ‐.25, *p* = .035)

**Conclusion:**

Findings demonstrate that people with more chronic illnesses may experience lower cognitive functioning, and racial and ethnic minorities may be particularly more vulnerable. Therefore, the prevention of chronic diseases plays an extremely important role in the cognitive health of older adults, specifically for vulnerable populations. Additionally, for racial and ethnic minorities, more emphasis may need to be placed on the prevention of chronic diseases. Policies aimed at preventing disease and promoting healthy lifestyles may also help improve cognitive functioning.

